# Perils of American Women

**Published:** 1885-08

**Authors:** 


PERILS OF AMERICAN WOMEN, or a Doctor's Talk with Maiden, Wife and Mother. By G. L. Austin, with a recommendatory letter from Mrs. Mary A. Livermore. Page 240. Boston: Lee & Shepard, publishers.

The author has made a mistake in writing this book in purely technical language when it is intended for the general public. It is unfortunate that he should have allowed the low fling made at gynecologists, which Mrs. Livermore makes in her recommendatory letter, which forms the frontispiece. She says: "I am especially thankful for Dr. Austin's disparaging words concerning the unclean army of gynecologists. I regard these specialists as a pestiferous set, and the bare mention of them is the same in its effect upon me as a red rag to a bull." The work of the publishers is excellent.

